# Protein restriction in CKD: an outdated strategy in the modern era

**DOI:** 10.1590/2175-8239-JBN-2024-PO03en

**Published:** 2025-02-10

**Authors:** Abdullah Bawazir, Joel M Topf, Swapnil Hiremath

**Affiliations:** 1 University of Ottawa, Ottawa Hospital, Department of Medicine, Division of Nephrology, Ottawa, ON, Canada.; 2 Ministry of National Guard Health Affairs, King Abdulaziz Medical City, Department of Medicine, Division of Nephrology, Riyadh, Saudi Arabia.; 3 Oakland University, William Beaumont School of Medicine, Rochester, Michigan, USA.

**Keywords:** Renal Insufficiency, Chronic, Dietary Proteins, Malnutrition, Quality of Life, Dietary Restrictions

## Abstract

Chronic kidney disease (CKD) management has traditionally emphasized dietary protein restriction to slow disease progression and delay end-stage renal disease (ESRD). However, evidence from trials questions the supposed efficacy of this approach and also highlights potential risks such as malnutrition and reduced quality of life. This review discusses the rational for protein restriction in CKD, critiques the existing evidence, and advocates for personalized care that focuses on nutritional adequacy and effective pharmacotherapy. Important advances in CKD treatment, including ACE inhibitors, SGLT2 inhibitors, and GLP-1 receptor agonists, are discussed to propose a comprehensive strategy that optimizes patient outcomes.

## Introduction

*‘All a low protein diet does is to shrink the patient down to the size of his kidneys.’* F. Parsons^1^.

Chronic kidney disease (CKD) is a significant global health issue, affecting more than 10% of the world population^2^. Traditional CKD management often includes dietary protein restriction to decrease glomerular filtration rate (GFR) and slow disease progression. Despite its popularity, data from clinical trial data have never been convincing and actually suggest that the benefits of protein restriction may pose risks, namely malnutrition and diminished quality of life. This review explores the history of protein restriction, analyzes recent research, and proposes that effective modern therapeutic approaches that prioritize patient-centered care should be given high priority.

## Historical Perspective and Guidelines

The recommendation for protein restriction in CKD stems from early 20th-century research showing that high protein intake led to increased GFR and heightened proteinuria in animal models^3,4^. The assumed mechanism behind the increase in GFR was afferent arteriolar vasodilation, likely mediated by nitric oxide^4,5^. These findings laid the groundwork for the longstanding belief that reducing protein intake could mitigate the progression of CKD by lowering GFR and reducing proteinuria. This became popular in the 1960s and 1970s, particularly with the Giordano-Giovannetti diet, which was based on physiological observations and case series rather than randomized trials^6^. Subsequent clinical trials have cast doubt on the effectiveness of this approach in humans, highlighting the need to re-evaluate dietary recommendations in CKD management.

The first documented human experiment on severe protein restriction was conducted nearly a century ago, in 1926^7^. The subject, a 28-year-old healthy medical student, was placed on a diet with minimal nitrogen content and rich in cornstarch, India gum, yeast, sugar, lard, and salt. This drastic reduction in protein intake led to extreme nausea, which was alleviated after 12 days of diet adjustment. Within three weeks, the student’s creatinine level decreased significantly from 0.74 mg/dL (65 μmol/L) to 0.41 mg/dL (36 μmol/L), indicating a reduction in muscle mass and/or an increase in kidney function. A 5% decrease in muscle strength was also observed. The experiment highlighted the challenging nature of such a restrictive diet, with the subject enthusiastic at the start but then became ‘alarmed’, ‘lost courage’, and abandoned the diet on the 25^th^ day.

Another 1964 study investigated the effects of two low-protein diets on six patients with chronic renal failure. Findings suggest that while a low-protein diet with high supplementation of essential amino acids may offer some advantages, such as easy adherence and slightly better nitrogen balance, these benefits are not sufficient to offset the risks associated with prolonged protein restriction, such as malnutrition and muscle loss, especially in patients with chronic renal failure^8^.

Over the years, guidelines from major organizations such as the Kidney Disease Improving Global Outcomes (KDIGO) and the Kidney Disease Outcomes Quality Initiative (KDOQI) have undergone significant changes, reflecting ongoing debates and new evidence in nephrology. Over the last two decades, despite little additional data, these guidelines have continued to evolve. At one end are the KDOQI (Kidney Disease Outcome and Quality Initiative) and the American Nutrition and Dietetics guidelines, which in 2020 recommended low and very low protein diet with supplemented keto/amino acids for patients with CKD stage 3 to 5 with a grade of 1A. At the other end, the UKKA (United Kingdom Kidney Association) guidelines recommend a normal dietary intake with no restriction. The KDIGO guidelines are in the middle and give a weak recommendation for a diet with 0.8 g/kg/day protein. Table 1 summarizes the different guidelines. It is instructive to examine the underlying evidence behind these wildly differing guidelines.

**Table 1:** Summary of different guidelines on protein intake recommendations in CKD patients

Organization	Year	CKD stage	Protein intake recommendations	Grade	Notes
KDOQI^9^	2000	GFR < 25 mL/min/1.73 m^2^	0.6g/kg/day	‘Evidence and Opinion’	For individuals who will not accept such a diet, an intake of up to 0.75 g/kg/day may be prescribed
Academy of Nutrition and Dietetics^10^	2010	GFR < 50 mL/min/1.73 m^2^ GFR < 20 mL/min/1.73 m^2^	0.6–0.8 g/kg/day 0.3–0.5 g/kg/day	1A 1A	Evidence rated as ‘Strong; Conditional’
KDIGO^11^	2012	Advanced CKD (GFR < 30 mL/min/1.73 m^2^)	0.8 g/kg/day Avoid high protein intake (>1.3 g/kg/day)	2B 2C	
UKKA^12^	2019	CKD stages 4–5	0.8–1 g/kg/day	1C	Recommended normal protein intake, no restriction
KDOQI and Academy of Nutrition and Dietetics^13^	2020	CKD stages 3–5 (metabolically stable)	0.55–0.60 g/kg/day (LPD) OR 0.3–0.4 g/kg/day (very LPD) with keto acid analogs to meet protein requirements	1A	Emphasizes risk reduction for ESRD/death and improving quality of life Evidence rated as ‘Strong, Conditional’
KDIGO^14^	2024	CKD stages 3–5 (metabolically stable)	Suggest 0.8 g/kg/day. Consider a very LPD (0.3– 0.4 g/kg body weight/d) with ketoacid analogs (up to 0.6 g/kg body weight/d) Avoid high protein intake (>1.3 g/kg body weight/d)	2C Ungraded practice point	Though guideline remained the same as 2012, the grade of evidence decreased for all the recommendations

Abbreviations – KDOQI: Kidney Disease Outcome Quality Initiative; KDIGO: Kidney Disease Improving Global Outcomes; UKKA: United Kingdom Kidney Association; GFR: glomerular filtration rate; CKD: chronic kidney disease; LPD: low protein diet; ESRD: end stage renal disease.

## Evidence from Randomized Controlled Trials

Randomized controlled trials (RCTs), including the landmark Modification of Diet in Renal Disease (MDRD) study^15^, have extensively investigated the effects of low-protein diets on the progression of CKD and are shown in Table 2. Early studies relied on changes in creatinine, creatinine clearance, or 1/creatinine analysis. However, changes in dietary protein intake can directly impact serum creatinine, and so all analyses of renal function are based on creatinine and should be interpreted very carefully, if at all considered in this discussion^21^.

**Table 2:** Landmark randomized, controlled trials of protein restriction in chronic kidney disease

Study	Participants	Diet groups	Findings	Critique and comments
Rosman et al.^16^	248 patients	0.4–0.6 g/kg/day vs. usual care	Significant decline in control group versus LPD group based on reciprocal of serum creatinine analysis	Use of creatinine for outcomes; Subjective acceptance of LPD was rated “bad” by one third of patients at 3 and 6 months
Ihle et al.^17^	72 (64 analyzed) patients	0.4 g/kg/day versus ‘usual diet’ with at least 0.75 g/kg/day protein	GFR slope slower with intervention and fewer people started dialysis (6 versus 27 percent)	Small difference was achieved (0.69 versus 0.85 g/kg/day) Single center trial, analysis was not intention-to-treat Greater weight loss with intervention
Locatelli et al.^18^	456 patients	0.6 g/kg/day vs. 1.0 g/kg/day	No significant difference in doubling serum creatinine or dialysis (p = 0.06)	Poor adherence (64 participants withdrew “lack of cooperation” for 58, “intolerance of low protein food” for 6)
Klahr et al.^15^	585 patients (Study 1), 255 patients (Study 2)	0.58 g/kg/day vs. 1.3 g/kg/day 0.28 g/kg/day vs. 0.58 g/kg/day	No significant benefit in slowing CKD progression based on GFR decline (primary outcome), or dialysis, or death	Excluded patients with diabetes; adherence to interventions was not perfect; 2 patients discontinued for malnutrition
Hansen et al.^19^	82 patients	0.6 g/kg/day vs. usual care	Cumulative incidence of ESRD or death: 30% in LPD group vs. 20% in usual care; GFR decline similar (p = 0.87)	Small sample size Tolerance or quality of life not reported
Cianciaruso et al.^20^	423 patients	0.55 g/kg/day vs. 0.8 g/kg/day	No significant difference in death or dialysis initiation (death: 12% vs. 11%; dialysis: 20% vs. 19%)	3 patients met criteria for protein-energy malnutrition

Abbreviations – GFR: glomerular filtration rate; LPD: low protein diet; ESRD: end stage renal disease.

**Figure 1:**
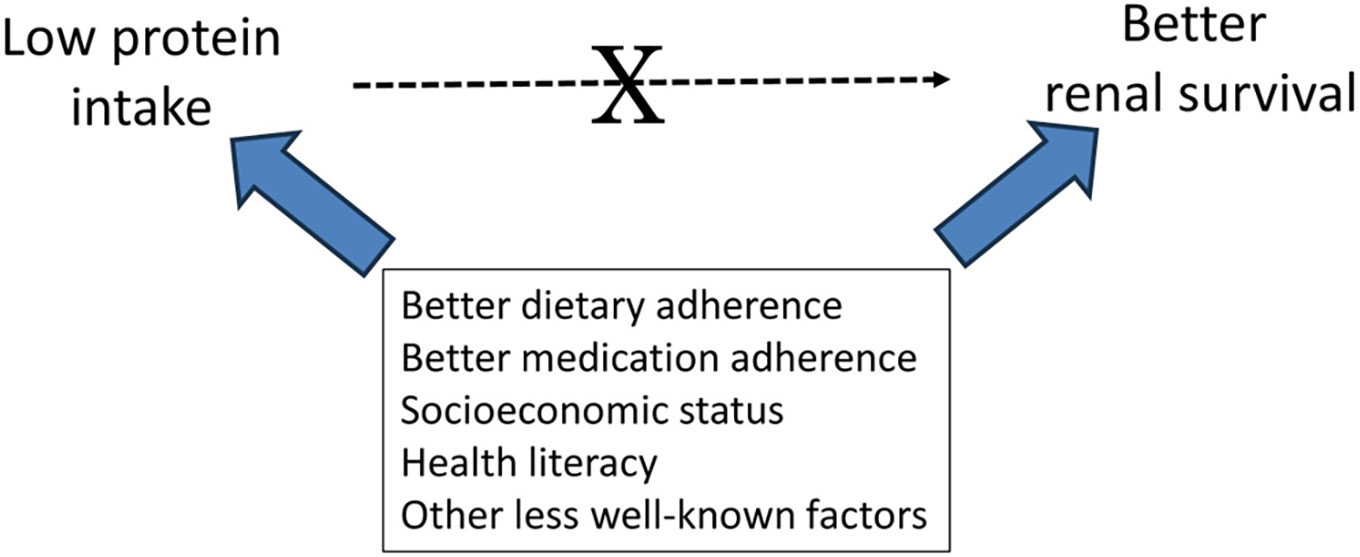
Reasons why observational studies of protein restriction can produce biased results.

The MDRD trial, conducted by Klahr et al.^15^, reported that there were no benefits in terms of slowing CKD progression or delaying the need for dialysis in patients with advanced CKD prescribed a low or very low protein diet. The MDRD study also had the advantage of using iothalamate clearance to measure GFR rather than serum creatinine or creatinine clearance. This method insulated the measurement from changes in muscle mass that can occur with lower protein intake. Of the several re-analyses and post-hoc studies that followed to explain these negative findings, many used the ‘achieved’ protein intake, analyzing subgroups based not on treatment assignment, but on actual protein intake. Such analyses effectively convert an RCT into a severely biased observational study. There are several reasons why people behave a certain way, and this behavior is often associated with outcomes in ways that cannot be teased out by regression or other statistical legerdemain (see Figure 1). As can be seen in Table 2, the trial data do not support any beneficial effect of protein restriction in CKD for renal survival. These findings are confirmed in a recent Cochrane review as well^22^.

## Challenges in Adherence and Nutrition

Protein-restricted diets are challenging, often leading to poor adherence and malnutrition risks (see quote at the beginning of the article). Patients frequently report difficulties maintaining low-protein diets, leading to nutritional deficiencies and related health complications. Additionally, there is growing skepticism about whether patients can realistically adhere to such stringent dietary restrictions over the long term. The complexities of everyday life, coupled with the social and psychological impact of a highly restrictive diet, often make it difficult for patients to consistently follow a low-protein regimen. The data supporting these conclusions can be found in the RCTs discussed before, and in the first study in the field, the acceptance of a low-protein diet was poor, with a third of the patients discontinuing the diet^16^. Another study attempted at reducing malnutrition in patients on a low-protein diet by using an isocaloric diet. The control diet was energy-dense and with non-protein nutrients, which was poorly tolerated. Nevertheless, the intervention group lost more weight and had lower serum transferrin levels, strongly suggestive of malnutrition^17^. Even in the MDRD study where keto analogs were used to decrease this risk, a few patients develop malnutrition. Most importantly, the longer term follow-up study of the MDRD study demonstrates the devastating harms of a low protein diet in patients with advanced CKD^23^. Of the 227 participants in study B of the MDRD, the mortality was almost twice as high with the very-low-protein diet (38.9% versus 23.3%, HR 1.92, 95% CI 1.15 – 3.20). These data are also supported by a recent large observational study including 8543 participants from Spain and Sweden, which reported a lower mortality with higher protein intake irrespective of the exact thresholds used, and findings were consistent in every subgroup examined^24^. Malnutrition is an important and difficult problem to address in patients with advanced CKD on dialysis, and there is little reason to compound it further by using an ineffective and nutritionally harmful intervention.

## Modern Therapeutic Approaches

**Figure 2:**
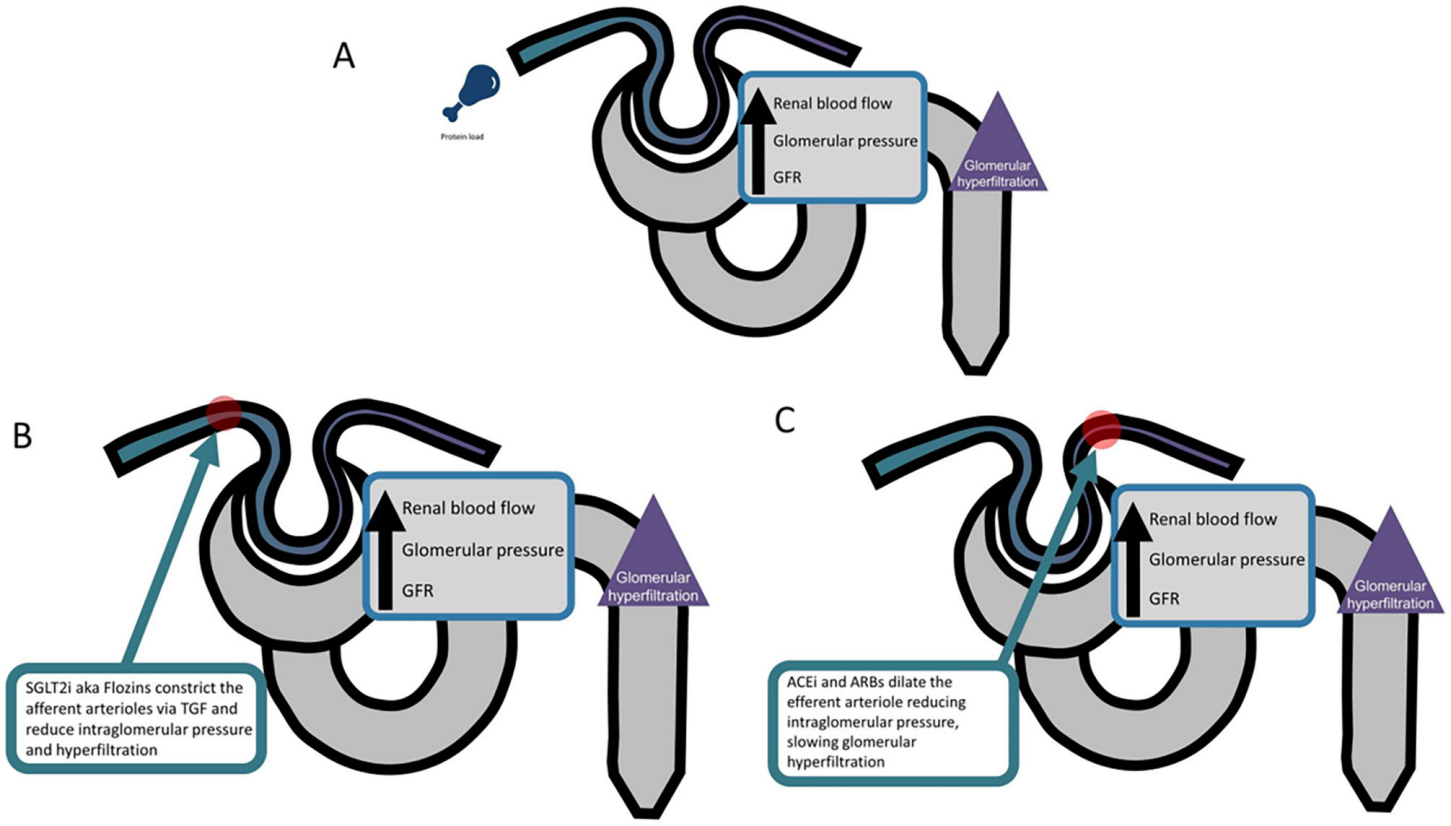
Schematic showing the effects of protein load (A), SGLT2 inhibitors (B), and RAS inhibitors (C) on the glomerulus and downstream consequences.

The studies and trials conducted in the heyday of the enthusiasm of the low protein diet followed the pathophysiology of progressive kidney disease as presented by the late Dr Brenner and blamed the hemodynamically mediated glomerular injury in the pathogenesis of progressive glomerular sclerosis in the elderly, renal ablation, and intrinsic renal disease on dietary protein intake^25^. While low-protein diets may reduce glomerular pressure and slow kidney damage by preventing the physiologic hyperfiltration that follows dietary protein intake (mediated by afferent arteriolar vasodilatation), pharmacological treatments such as angiotensin-converting enzyme inhibitors (ACE inhibitors), angiotensin-II receptor blockers (ARBs), and sodium-glucose cotransporter 2 inhibitors (SGLT2 inhibitors or flozins) achieve similar, if not superior, effects by dilating both the afferent and efferent arterioles (see Figure 2)^26,27^.

The efficacy of renin-angiotensin system (RAS) inhibitors and SGLT2 inhibitors is well-supported by substantial clinical evidence, making them a viable and often preferred alternative to the dietary restrictions associated with low-protein diets. For instance, ACE inhibitors have been shown to reduce proteinuria and slow the progression of CKD by modulating the renin-angiotensin-aldosterone system, thereby offering better renal protection than dietary changes alone. In a recent meta-analysis examining over 18 trials, ACE inhibitors (ACEi) and ARBs were associated with a significantly lower risk of kidney failure requiring renal replacement therapy (RRT), with an adjusted hazard ratio of 0.66 (95% CI, 0.55 to 0.79)^28^. Similarly, SGLT2 inhibitors, such as empagliflozin, canagliflozin, and dapagliflozin, have demonstrated significant benefits in reducing intraglomerular pressure, improving renal outcomes, and reducing cardiovascular events in patients with CKD, irrespective of diabetic status. Meta-analyses have shown a 15% (*p* < .0001) reduction in all-cause mortality and 16% (*p* = .0006) reduction in cardiovascular mortality, with a significant decrease in need for dialysis and heart failure hospitalizations^29^.

Adding to these traditional pharmacotherapies, new developments in CKD management include glucagon-like peptide-1 (GLP-1) receptor agonists. These agents not only provide significant renal benefits but also offer cardiovascular advantages, which are critical for patients with CKD who often have a high risk of cardiovascular events. The recent FLOW trial highlighted the efficacy of GLP-1 receptor agonists in reducing the risk of renal events and slowing the decline of GFR in patients with type 2 diabetes and CKD^30^. This marks a significant advance in CKD therapy that improves and complements existing treatments such as ACE inhibitors and SGLT2 inhibitors.

By using these pharmacological interventions, patients can achieve more consistent and predictable management of kidney health, alleviating the need for strict dietary compliance and reducing the risk of malnutrition and its associated complications. This approach not only simplifies the management of CKD, but also aligns with current clinical practices that emphasize the importance of individualized and evidence-based treatment strategies. The early trials with protein restriction were performed in the era when even the use of RAS inhibitors was just emerging, and it is largely unexplored whether an additional a low-protein diet has any complementary effects when added to the background of contemporary pharmacotherapy, especially considering how poorly these diets performed against the standard of care of the 1980s and 90s.

## Cost Considerations

The purported low cost of low-protein diets is sometimes touted as a reason to use these diets. However, any cost-related benefit of low protein diets is meaningless since the intervention itself is not an effective one. Secondly, the costs of a low-protein diet are not insignificant. Changing diet is not always cost-neutral. Additionally, the means to prevent malnutrition require the use of amino acid and keto acid analogs, which are prohibitively expensive, with costs that approach that of dialysis. In contrast, almost all RAS inhibitors are generic worldwide and cost only a few cents per day. Certain SGLT2 inhibitors have already become generic, and others will soon in the coming years. Kidney organizations and nephrology professionals should focus their attention on the implementation of effective therapies, rather than explore ineffective diets from the 20^th^ century.

## Quantity or Quality of Protein

Going beyond the quantity of protein, the type of dietary protein consumed may play a significant role in CKD outcomes. A Tehran Lipid and Glucose Study found that higher consumption of red and processed meats increases the risk of CKD. Specifically, the odds ratio (OR) for CKD was 1.73 (95% CI: 1.33–2.24) for high red meat intake and 1.99 (95% CI: 1.54–2.56) for processed meat. Conversely, plant-based proteins like legumes and nuts were associated with a lower risk, reducing CKD incidence by 19% (OR: 0.81; 95% CI: 0.68–0.97) and 16% (OR: 0.84; 95% CI: 0.74–0.95), respectively. The caveat here is that these are again observational studies, susceptible to confounding and selection bias (see Figure 1), and these observations need to be confirmed in properly designed RCTs before being incorporated into clinical guidance^31^.

## Conclusion

In summary, the current evidence is clearly and categorically against routine protein restriction in CKD management. The challenges with adherence, the risk of malnutrition, and the availability of effective pharmacotherapies indicate that protein restriction is an outdated approach. Modern CKD management should prioritize evidence-based pharmacotherapies and ensure adequate nutritional support to improve patient outcomes and quality of life. Future guidelines should reflect the latest research and provide more personalized dietary recommendations that consider the patient’s overall health and well-being.
