# Supplementary material for: Protein restriction in CKD: an outdated strategy in the modern era
Source: J Bras Nefrol. 2025 Feb 10;47(1):e2024PO03. doi: 10.1590/2175-8239-JBN-2024-PO03en (PMC11813190; doi:10.1590/2175-8239-JBN-2024-PO03en)
Supplement: Translation

# Translation: Restrição proteica na DRC: uma estratégia obsoleta na era moderna

**DOI:** 10.1590/2175-8239-JBN-2024-PO03pt

**Authors:** Abdullah Bawazir, Joel M Topf, Swapnil Hiremath

**Affiliations:** 1 University of Ottawa, Ottawa Hospital, Departamento de Medicina, Divisão de Nefrologia, Ottawa, ON, Canadá.; 2 Ministry of National Guard Health Affairs, King Abdulaziz Medical City, Departamento de Medicina, Divisão de Nefrologia, Riyadh, Arábia Saudita.; 3 Oakland University, William Beaumont School of Medicine, Rochester, Michigan, EUA.

**Keywords:** Insuficiência Renal, Crônica, Proteínas Dietéticas, Desnutrição, Qualidade de Vida, Restrições Dietéticas

## Abstract

Tradicionalmente, o manejo da doença renal crônica (DRC) tem enfatizado a restrição dietética de proteínas para retardar a progressão da doença e atrasar a doença renal em estágio terminal (DRET). No entanto, evidências de estudos clínicos questionam a suposta eficácia dessa abordagem e também destacam potenciais riscos, como desnutrição e redução da qualidade de vida. Esta revisão discute os fundamentos para a restrição proteica na DRC, apresenta críticas às evidências existentes e defende um cuidado personalizado que priorize a adequação nutricional e uma farmacoterapia eficaz. Avanços importantes no tratamento da DRC, incluindo inibidores da ECA, inibidores de SGLT2 e agonistas do receptor de GLP-1, são discutidos com o objetivo de propor uma estratégia abrangente que otimize os desfechos dos pacientes.

## Introdução

*‘Tudo o que uma dieta de baixa proteína faz é reduzir o paciente ao tamanho de seus rins.’* F. Parsons^1^.

A doença renal crônica (DRC) é um problema de saúde global significativo, afetando mais de 10% da população mundial^2^. O manejo tradicional da DRC geralmente inclui a restrição dietética de proteínas para diminuir a taxa de filtração glomerular (TFG) e retardar a progressão da doença. Apesar de sua popularidade, os dados de ensaios clínicos nunca foram convincentes e, na verdade, sugerem que os benefícios da restrição proteica podem apresentar riscos, como desnutrição e perda da qualidade de vida. Esta revisão explora o histórico da restrição proteica, analisa pesquisas recentes e propõe que abordagens terapêuticas modernas e eficazes, que priorizem o cuidado centrado no paciente, devam receber alta prioridade.

## Perspectiva Histórica e Diretrizes

A recomendação para restrição proteica na DRC tem origem em pesquisas do início do século XX, que demonstraram que a ingestão elevada de proteínas levava ao aumento da TFG e à elevação da proteinúria em modelos animais^3,4^. O mecanismo presumido por trás do aumento da TFG foi a vasodilatação arteriolar aferente, provavelmente mediada pelo óxido nítrico^4,5^. Esses achados estabeleceram as bases para a crença de longa data de que a redução da ingestão proteica poderia mitigar a progressão da DRC, ao diminuir a TFG e reduzir a proteinúria. Isso se tornou popular nas décadas de 1960 e 1970, especialmente com a dieta Giordano-Giovannetti, que se baseava em observações fisiológicas e séries de casos, em vez de estudos randomizados^6^. Ensaios clínicos posteriores colocaram em dúvida a eficácia dessa abordagem em seres humanos, destacando a necessidade de reavaliar as recomendações dietéticas no manejo da DRC.

O primeiro experimento documentado em humanos sobre a restrição severa de proteínas foi realizado há quase um século, em 1926^7^. O indivíduo, um estudante de medicina saudável de 28 anos, foi submetido a uma dieta com teor mínimo de nitrogênio e rica em amido de milho, goma guar, levedura, açúcar, banha e sal. Essa redução drástica na ingestão de proteínas levou a náuseas extremas, que foram aliviadas após 12 dias de ajuste da dieta. Em três semanas, o nível de creatinina do estudante diminuiu significativamente de 0,74 mg/dL (65 μmol/L) para 0,41 mg/dL (36 μmol/L), indicando uma redução na massa muscular e/ou um aumento na função renal. Também foi observada uma redução de 5% na força muscular. O experimento destacou a natureza desafiadora de uma dieta tão restritiva, com um sujeito inicialmente entusiasmado, mas que posteriormente ficou “alarmado”, “perdeu a coragem” e abandonou a dieta no 25º dia.

Outro estudo de 1964 investigou os efeitos de duas dietas com baixo teor de proteína em seis pacientes com insuficiência renal crônica. Os achados sugerem que, embora uma dieta de baixa proteína com alta suplementação de aminoácidos essenciais possa oferecer algumas vantagens, como fácil adesão e equilíbrio nitrogenado ligeiramente superior, esses benefícios não são suficientes para compensar os riscos associados à restrição proteica prolongada, como desnutrição e perda muscular, especialmente em pacientes com insuficiência renal crônica^8^.

Ao longo dos anos, as diretrizes das principais organizações, como KDIGO (*Kidney Disease Improving Global Outcomes*) e KDOQI (*Kidney Disease Outcomes Quality Initiative*), passaram por alterações significativas, refletindo debates contínuos e novas evidências na nefrologia. Nas últimas duas décadas, apesar de poucos dados adicionais, essas diretrizes continuaram a evoluir. Em um extremo estão as diretrizes do KDOQI (*Kidney Disease Outcome and Quality Initiative*) e da *American Nutrition and Dietetics*, que, em 2020, recomendaram dietas de baixa e muito baixa ingestão proteica com suplementação de ceto/aminoácidos para pacientes com DRC em estágio 3 a 5 com grau 1A. No outro extremo, as diretrizes da UKKA (*United Kingdom Kidney Association*) recomendam uma ingestão dietética normal, sem restrições. As diretrizes do KDIGO encontram-se no meio termo e fornecem uma recomendação fraca para uma dieta com 0,8 g/kg/dia de proteína. A Tabela 1 resume as diferentes diretrizes. É instrutivo examinar as evidências subjacentes por trás dessas diretrizes extremamente diferentes.

**Tabela 1:** Resumo das diferentes diretrizes sobre recomendações de ingestão de proteínas em pacientes com DRC

Organização	Ano	Estágio da DRC	Recomendações de ingestão proteica	Grau	Observações
KDOQI^9^	2000	TFG < 25 mL/min/ 1,73 m^2^	0,6 g/kg/dia	‘Evidência e Opinião’	Para indivíduos que não aceitam tal dieta, pode ser prescrita uma ingestão de até 0,75 g/kg/dia
Academia de Nutrição e Dietética^10^	2010	TFG < 50 mL/min/1,73 m^2^ TFG < 20 mL/min/1,73 m^2^	0,6–0,8 g/kg/dia 0,3–0,5 g/kg/dia	1A 1A	Evidência classificada como ‘Forte; Condicional’
KDIGO^11^	2012	DRC avançada (TFG < 30 mL/min/1,73 m^2^)	0,8 g/kg/dia Evitar alta ingestão de proteínas (>1,3 g/kg/dia)	2B 2C	
UKKA^12^	2019	DRC estágios 4–5	0,8–1 g/kg/dia	1C	Recomendada ingestão normal de proteínas, sem restrições
KDOQI e Academia de Nutrição e Dietética^13^	2020	DRC estágios 3–5 (metabolicamente estável)	0,55–0,60 g/kg/dia (LPD) OU 0,3–0,4 g/kg/dia (dieta com teor muito baixo de proteína) com análogos de cetoácidos para atender às necessidades de proteína	1A	Enfatiza redução do risco para DRET/óbito e melhoria da qualidade de vida Evidência classificada como ‘Forte, Condicional’
KDIGO^14^	2024	DRC estágios 3–5 (metabolicamente estável)	Sugerir 0,8 g/kg/dia. Considerar dieta com teor muito baixo de proteína (0,3–0,4 g/kg de peso corporal/d) com análogos de cetoácidos (até 0,6 g/kg de peso corporal/d) Evitar alta ingestão de proteínas (>1,3 g/kg de peso corporal/d)	2C Ponto de prática não avaliado	Embora a diretriz tenha permanecido a mesma de 2012, o grau de evidência diminuiu para todas as recomendações

Abreviaturas – KDOQI: *Kidney Disease Outcome Quality Initiative;* KDIGO: *Kidney Disease Improving Global Outcomes;* UKKA: *United Kingdom Kidney Association;* TFG: taxa de filtração glomerular; DRC: doença renal crônica; LPD: dieta de baixa proteína; DRET: doença renal em estágio terminal.

## Evidências de Ensaios Clínicos Randomizados

Ensaios clínicos randomizados (ECRs), incluindo o estudo de referência *Modification of Diet in Renal Disease* (MDRD)^15^, investigaram amplamente os efeitos de dietas com baixo teor de proteína na progressão da DRC, conforme demonstrado na Tabela 2. Estudos iniciais basearam-se em alterações na creatinina, *clearance* de creatinina ou análise de 1/creatinina. No entanto, alterações na ingestão dietética de proteínas podem impactar diretamente os níveis de creatinina sérica. Portanto, todas as análises da função renal são baseadas na creatinina e devem ser interpretadas com muito cuidado caso sejam consideradas nessa discussão^21^.

**Table 2:** Ensaios clínicos randomizados e controlados de referência sobre restrição proteica na doença renal crônica

Estudo	Participantes	Grupos de dieta	Achados	Críticas e comentários
Rosman et al.^16^	248 pacientes	0,4–0,6 g/kg/dia vs. tratamento usual	Declínio significativo no grupo controle versus grupo LPD com base na recíproca da análise de creatinina sérica	Uso de creatinina para desfechos; a aceitação subjetiva da LPD foi classificada como “ruim” por um terço dos pacientes aos 3 e 6 meses
Ihle et al.^17^	72 pacientes (64 analisados)	0,4 g/kg/dia versus ‘dieta usual’ com pelo menos 0,75 g/kg/dia de proteína	O declínio da TFG foi mais lento com a intervenção e menos pessoas iniciaram diálise (6 versus 27%)	Pequena diferença alcançada (0,69 versus 0,85 g/kg/dia) Estudo de centro único, a análise não foi por intenção de tratar Maior perda de peso com intervenção
Locatelli et al.^18^	456 pacientes	0,6 g/kg/dia vs. 1,0 g/kg/dia	Nenhuma diferença significativa na duplicação da creatinina sérica ou na diálise (p = 0,06)	Baixa adesão (64 participantes desistiram: 58 por “falta de cooperação” e 6 por “intolerância a alimentos com baixo teor de proteína”)
Klahr et al.^15^	585 pacientes (Estudo 1), 255 pacientes (Estudo 2)	0,58 g/kg/dia vs. 1,3 g/kg/dia 0,28 g/kg/dia vs. 0,58 g/kg/dia	Nenhum benefício significativo em retardar a progressão da DRC com base no declínio da TFG (desfecho primário), diálise ou óbito	Pacientes com diabetes foram excluídos; a adesão às intervenções não foi perfeita; 2 pacientes foram descontinuados por desnutrição
Hansen et al.^19^	82 pacientes	0,6 g/kg/dia vs. tratamento usual	Incidência cumulativa de DRET ou óbito: 30% no grupo LPD vs. 20% no tratamento usual; declínio da TFG semelhante (p = 0,87)	Tamanho pequeno da amostra Tolerância ou qualidade de vida não relatadas
Cianciaruso et al.^20^	423 pacientes	0,55 g/kg/dia vs. 0,8 g/kg/dia	Nenhuma diferença significativa em óbito ou início de diálise (óbito: 12% vs. 11%; diálise: 20% vs. 19%)	3 pacientes preencheram os critérios para desnutrição proteico-energética

Abreviaturas – TFG: taxa de filtração glomerular; LPD: dieta de baixa proteína; DRET: doença renal em estágio terminal.

O estudo MDRD, conduzido por Klahr et al.^15^, relatou que não houve benefícios em termos de retardar a progressão da DRC ou adiar a necessidade de diálise em pacientes com DRC avançada com prescrição de uma dieta com baixo ou muito baixo teor proteico. O estudo MDRD também teve a vantagem de utilizar o *clearance* de iotalamato para medir a TFG em vez da creatinina sérica ou do *clearance* de creatinina. Esse método isolou a medição de alterações na massa muscular que podem ocorrer com uma ingestão reduzida de proteínas. Das várias reanálises e estudos post-hoc que se seguiram para explicar esses achados negativos, muitos utilizaram a ingestão de proteínas “alcançada”, analisando subgrupos com base não na atribuição de tratamento, mas na ingestão real de proteínas. Essas análises efetivamente transformam um ECR em um estudo observacional com viés grave. Existem diversas razões pelas quais as pessoas se comportam de uma determinada maneira, e esse comportamento frequentemente está associado a desfechos de uma forma que não pode ser deduzida por meio de regressão ou outros artifícios estatísticos (ver Figura 1). Como pode ser visto na Tabela 2, os dados dos estudos não sustentam qualquer efeito benéfico da restrição proteica na DRC para a sobrevida renal. Esses achados também são confirmados em uma recente revisão produzida pela Cochrane^22^.

**Figura 1:** Razões pelas quais estudos observacionais sobre restrição proteica podem produzir resultados tendenciosos.

## Desafios na Adesão e na Nutrição

As dietas com restrição proteica são desafiadoras, muitas vezes levando a uma baixa adesão e a riscos de desnutrição (ver citação no início do artigo). Os pacientes frequentemente relatam dificuldades em manter dietas de baixa proteína, o que resulta em deficiências nutricionais e complicações de saúde relacionadas. Além disso, há um ceticismo crescente sobre a possibilidade de os pacientes aderirem de maneira realista a restrições dietéticas tão rigorosas em longo prazo. As complexidades da vida cotidiana, aliadas ao impacto social e psicológico de uma dieta altamente restritiva, muitas vezes tornam difícil para os pacientes seguirem de maneira consistente um regime de baixo teor de proteína. Os dados que sustentam essas conclusões podem ser encontrados nos ECRs discutidos anteriormente e, no primeiro estudo na área, a aceitação de uma dieta com baixo teor de proteína foi baixa, com um terço dos pacientes interrompendo a dieta^16^. Outro estudo tentou reduzir a desnutrição em pacientes com uma dieta de baixa proteína utilizando uma dieta isocalórica. A dieta controle apresentava alta densidade energética com nutrientes não proteicos, o que foi mal tolerado. No entanto, o grupo de intervenção perdeu mais peso e apresentou níveis mais baixos de transferrina sérica, o que sugere fortemente a desnutrição^17^. Mesmo no estudo MDRD, em que os análogos de ceto foram utilizados para reduzir esse risco, alguns pacientes desenvolveram desnutrição. Mais importante, o estudo de acompanhamento de longo prazo do estudo MDRD demonstra os danos devastadores de uma dieta de baixa proteína em pacientes com DRC avançada^23^. Dos 227 participantes no estudo B do MDRD, a mortalidade foi quase duas vezes maior com a dieta de proteína muito baixa (38,9% versus 23,3%; HR 1,92; IC 95% 1,15–3,20). Esses dados também são corroborados por um grande estudo observacional recente incluindo 8.543 participantes da Espanha e da Suécia, que relatou menor mortalidade com maior ingestão de proteína, independentemente dos limites exatos utilizados, e os achados foram consistentes em todos os subgrupos examinados^24^. A desnutrição é um problema importante e de difícil abordagem em pacientes com DRC avançada submetidos à diálise, e não há motivo para agravá-la ainda mais com o uso de uma intervenção ineficaz e prejudicial do ponto de vista nutricional.

## Abordagens Terapêuticas Modernas

Os estudos e ensaios realizados no auge do entusiasmo pela dieta com baixo teor de proteína seguiram a fisiopatologia da doença renal progressiva conforme apresentada pelo falecido Dr. Brenner e atribuíram a lesão glomerular mediada hemodinamicamente na patogênese da esclerose glomerular progressiva em idosos, ablação renal e doença renal intrínseca à ingestão dietética de proteína^25^. Embora as dietas com baixo teor proteico possam reduzir a pressão glomerular e retardar o dano renal ao prevenir a hiperfiltração fisiológica que se segue à ingestão de proteínas na dieta (mediada pela vasodilatação das arteríolas aferentes), tratamentos farmacológicos – como os inibidores da enzima conversora de angiotensina (inibidores da ECA), bloqueadores do receptor de angiotensina-II (BRAs) e inibidores do cotransportador sódio-glicose 2 (inibidores de SGLT2 ou flozinas) - alcançam efeitos semelhantes, se não superiores, ao dilatar as arteríolas aferentes e eferentes (ver Figura 2)^26,27^.

**Figura 2:** Esquema mostrando os efeitos da carga proteica (A), inibidores de SGLT2 (B) e inibidores do SRA (C) no glomérulo e consequências posteriores.

A eficácia dos inibidores do sistema renina-angiotensina (SRA) e dos inibidores de SGLT2 é bem sustentada por evidências clínicas substanciais, o que os torna uma alternativa viável e muitas vezes preferida às restrições alimentares associadas às dietas de baixa proteína. Por exemplo, os inibidores da ECA demonstraram reduzir a proteinúria e retardar a progressão da DRC por meio da modulação do sistema renina-angiotensina-aldosterona, oferecendo, assim, uma proteção renal superior às alterações dietéticas isoladas. Em uma meta-análise recente que examinou mais de 18 estudos, os inibidores da ECA (iECA) e os BRAs foram associados a um risco significativamente menor de insuficiência renal com necessidade de terapia renal substitutiva (TRS), com uma razão de risco ajustada de 0,66 (IC 95%, 0,55 a 0,79)^28^. Da mesma forma, os inibidores de SGLT2, como empagliflozina, canagliflozina e dapagliflozina, demonstraram benefícios significativos na redução da pressão intraglomerular, na melhora dos desfechos renais e na redução de eventos cardiovasculares em pacientes com DRC, independentemente do estado diabético. Meta-análises demonstraram uma redução de 15% (p < 0,0001) na mortalidade por todas as causas e uma redução de 16% (p = 0,0006) na mortalidade cardiovascular, com uma diminuição significativa na necessidade de diálise e hospitalizações por insuficiência cardíaca^29^.

Além dessas farmacoterapias tradicionais, novos progressos no manejo da DRC incluem agonistas do receptor do peptídeo semelhante ao glucagon-1 (GLP-1). Esses agentes não apenas proporcionam benefícios renais significativos, mas também oferecem vantagens cardiovasculares, que são fundamentais para pacientes com DRC, os quais frequentemente apresentam um alto risco de eventos cardiovasculares. O recente estudo FLOW destacou a eficácia dos agonistas do receptor de GLP-1 na redução do risco de eventos renais e na diminuição do declínio da TFG em pacientes com diabetes tipo 2 e DRC^30^. Isso representa um avanço significativo na terapia da DRC que melhora e complementa os tratamentos existentes, como os inibidores da ECA e os inibidores de SGLT2.

Ao utilizar essas intervenções farmacológicas, os pacientes podem alcançar um manejo mais consistente e previsível da saúde renal, aliviando a necessidade de cumprimento rigoroso da dieta e reduzindo o risco de desnutrição e suas complicações associadas. Essa abordagem não apenas simplifica o manejo da DRC, mas também se alinha às práticas clínicas atuais que enfatizam a importância de estratégias de tratamento individualizadas e baseadas em evidências. Os primeiros estudos com restrição proteica foram realizados na época em que até mesmo o uso de inibidores do SRA estava somente começando, e ainda não foi amplamente explorado se uma dieta adicional com baixo teor de proteína tem algum efeito complementar quando adicionada ao contexto da farmacoterapia contemporânea, especialmente considerando quão ruim foi o desempenho dessas dietas em relação ao padrão de tratamento das décadas de 1980 e 1990.

## Considerações Sobre Custos

O suposto baixo custo das dietas de baixa proteína é, às vezes, apresentado como uma razão para utilizá-las. No entanto, qualquer benefício relacionado ao custo de dietas com baixo teor de proteína não tem sentido, uma vez que a intervenção em si não é eficaz. Em segundo lugar, os custos de uma dieta de baixa proteína não são insignificantes. Mudar a dieta nem sempre representa um custo neutro. Além disso, os meios para prevenir a desnutrição exigem o uso de análogos de aminoácidos e cetoácidos, que são proibitivamente caros, com custos que se aproximam aos da diálise. Em contrapartida, quase todos os inibidores do SRA são genéricos no mundo todo e custam apenas alguns centavos por dia. Certos inibidores de SGLT2 já se tornaram genéricos, e outros o serão nos próximos anos. As organizações renais e os profissionais de nefrologia devem concentrar seus esforços na implementação de terapias eficazes, em vez de explorar dietas ineficazes do século XX.

## Quantidade ou Qualidade da Proteína

Para além da quantidade de proteína, o tipo de proteína dietética consumida pode desempenhar um papel significativo nos desfechos da DRC. O estudo *Tehran Lipid and Glucose* revelou que o maior consumo de carnes vermelhas e processadas aumenta o risco de DRC. Especificamente, o *odds ratio* (OR) para DRC foi de 1,73 (IC 95%: 1,33–2,24) para alta ingestão de carne vermelha e 1,99 (IC 95%: 1,54–2,56) para carne processada. Por outro lado, as proteínas de origem vegetal, como legumes e nozes, foram associadas a um risco menor, reduzindo a incidência de DRC em 19% (OR: 0,81; IC 95%: 0,68–0,97) e 16% (OR: 0,84; IC 95%: 0,74–0,95), respectivamente. A ressalva aqui é que esses são novamente estudos observacionais, suscetíveis a vieses de confusão e seleção (ver Figura 1), e essas observações precisam ser confirmadas em ECRs adequadamente desenhados antes de serem incorporadas à orientação clínica^31^.

## Conclusão

Em resumo, as evidências atuais são clara e categoricamente contrárias à restrição proteica de rotina no manejo da DRC. Os desafios com a adesão, o risco de desnutrição e a disponibilidade de farmacoterapias eficazes indicam que a restrição proteica é uma abordagem obsoleta. O manejo moderno da DRC deve priorizar farmacoterapias baseadas em evidências e garantir o suporte nutricional adequado para melhorar os desfechos e a qualidade de vida dos pacientes. As diretrizes futuras devem refletir as pesquisas mais recentes e fornecer recomendações dietéticas mais personalizadas, que considerem a saúde e o bem-estar geral do paciente.
